# Prevalence and risk factors of bovine schistosomiasis in Northwestern Ethiopia

**DOI:** 10.1186/s12917-018-1757-9

**Published:** 2019-01-07

**Authors:** Abebe Yihunie, Befikadu Urga, Getachew Alebie

**Affiliations:** 1 College of Veterinary Medicine, Jigjiga University, P.O.Box- 1020, Jijiga, Ethiopia; 2 Department of Biology, Jigjiga University, P.O. Box- 1020, Jijiga, Ethiopia

**Keywords:** Prevalence, Risk factors, Bovine schistosomiasis, South Achefer, Ethiopia

## Abstract

**Background:**

Parasitic diseases remain major bottle neck to livestock development in developing nations. The objective of this study was to determine the prevalence and associated risk factors of Bovine Schistosomiasis (BS) in South Achefer District, northwestern Ethiopia.

**Methods:**

A cross-sectional copro-parasitological and observational study was conducted in South Achefer district from October, 2015 to April, 2016. Faecal samples were collected from 360 randomly selected cattle for coprological examination of *Schistosoma* eggs using sedimentation technique. The geographical origin (kebele), signalment (breed, sex and age) and body condition of study animals were recorded as independent variables.

**Results:**

Of the total of 360 faecal samples examined, 80 (22.2%) were found positive for *Schistosoma bovis* eggs. Prevalence of BS showed significant variability amongst study kebele’s (*p* = 0.000) as well as between different breeds (*p* = 0.009), sexes (*p* = 0.030) and body condition groups (*p* = 0.000) of study animals. Compared to Gedema kebele, risk of *Schistosomia bovis* infection was significantly higher (*p* < 0.05) in Ahurie kebele (95% CI OR, 1.497–6.680) and lower in Kar kebele (95% CI OR, 0.069–0.507). Meanwhile, risk of BS was significantly higher in cattle with poor body condition (95% CI OR, 3.171–15.652) as compared to that exhibiting good body condition. Local breed (95% CI OR, 1.282–5.102) and female (95% CI OR, 1.018–3.634) cattle showed considerably higher risk of infection than crossbred and male cattle, respectively.

**Conclusion:**

Overall, agro-ecological, genotypic and sexual factors were important in determining prevalence of BS which had negative association with the nutritional status of cattle. Current and parallel prior observations underscore a need for careful consideration of the disease and its epidemiological drivers in genetic improvement programs and routine health management practices.

## Background

Schistosomiasis caused by trematodes of the genus *Schistosoma*, is an economically and medically important parasitic disease of man and animals [1]. The disease is responsible for considerable economic losses in livestock industry, mainly through mortality, reduced fertility and productivity, stunted growth [1–3].

The major schistosome species of veterinary importance include *Schistosoma bovis*, *S. mattheei, S. intercalatum, S. spindale*, *S. nasalis, S. indicum, S. hippopotami* and *S. rohhaini* [2, 4]. Snails belonging to *Bulinus* and *Physopsis* genera*,* serve as intermediate hosts of bovine schistosomes, potentially determining distribution of BS in different parts of the world [5]. *S. bovis* is the commonest species in Africa and Mediterranean Europe [6] whereas *S. spindale, S. indicum* and *S. nasalis* were reported as major cause of BS in Asia [4]. Infestation with bovine schistosome parasites is usually associated with grazing in wet land and drinking from the snail infested watering places [7].

In Ethiopia, *S. bovis* is known to be the main cause of BS which is recognized as an important bottleneck to livestock development. The disease is focally distributed in northern, eastern, south western and central parts of the nation [8]. Available evidences reveal that its prevalence in the country ranges from 1.5 to 37.3% [9–16]. This study aimed to estimate the prevalence of BS and identify associated risk factors in north western Ethiopia.

## Methods

### Study area

The study was conducted in South Achefer district, North-western Ethiopia. The district is one of the thirteen districts found in West Gojjam Administrative Zone located 60 km south-west of Bahir Dar town, the capital city of Amhara Regional State. According to the district agricultural office sources, the total geographical area of South Achefer district is about 118,228 ha. The total human population of the district is about 148,974; of which 134,447 or 90.2% live in rural areas and 14,528 or 9.8% of the population is urban resident [17]. The estimated livestock population of the district is 53,612 cattle, 80,868 sheep and goats, 22,375 equines, 16,721 bee colonies, 74,689 poultry and 16,684 other domestic animals [18]. South Achefer district is located at latitude of 11° 21 32”N and longitude of 36°57′42″ E. Altitudes in the district range from 1500 to 2500 m above mean sea level. The mean annual rain fall ranges from 1450 to 1594 mm with average annual temperature of 26.8 °C.

According to the Amhara Design and Supervision Works Enterprise (ADSWE) Animal Health Assessment report in different districts along Tana-basin, liver fluke (Fascioliasis) and schistosomiasis were ranked as top two livestock health problems in South Achefer district in terms of their severity, economic importance and incidence [19]. The report revealed these diseases to be very series problem in permanent wet-lands of the district causing poor weight gain, death, poor milk yield, poor feed conversion and poor reproductive capacity.

### Study design

A cross-sectional copro-parasitological and observational study was conducted to assess the prevalence and associated risk factors of BS in South Achefer district between October 2015 and April 2016. All cattle (53,612 heads) of different breeds, sex and age groups in the district were enrolled in the study. Two-stage semi-random sampling was applied for selecting cattle for the field survey. In the first stage, study kebeles (Ahirie, Afrefida, Kar and Gedema) were selected based on practical logistic and demographic considerations. In the second stage, every 5th farm household encountered during field survey was systematically selected and then cattle were randomly selected for the study.

### Sample size determination

Sample size (n) was determined according to Thrusfield [20], with 95% confidence level (*z* = 1.96), 5% desired absolute precision (d) and 24.3% expected prevalence (pexp) [11].

$$ \mathrm{i}.\mathrm{e}.\kern0.62em \mathrm{n}=\frac{{\mathrm{z}}^2\mathrm{pexp}\kern0.5em \left(1\hbox{--} \mathrm{pexp}\right)}{{\mathrm{d}}^2} $$

This gave a sample size of 283 heads of cattle. To generate reliable data outputs and to increase the precision, the sample size was increased to 360 heads of cattle.

### Observation

The geographical origin (kebele), signalment (breed, sex and age) and body condition of study animals were noted through visual inspection and owner’s interview. Breed of cattle was categorized as local (Fogera breed) or cross-bred (Local - Holstein Friesian). Age of the cattle was categorized following the methods of Pope [21], and Ferguson [22] respectively whereas body condition was subjectively rated as good (no visible bony prominences i.e. rib cage and tail base), medium (outline of bony prominences visible) or poor (grossly prominent bony prominences) based on visual assessment.

### Coprological examination

Fresh faecal samples were directly collected from the rectum of cattle in the field using gloved hand. The collected sample was preserved in 10% formalin in clean and labelled screw cap universal bottle to prevent hatching of miracidia before reaching laboratory to diagnose within 24 h of collection. It was placed in ice box and transported to Durbete veterinary clinic parasitology laboratory. Then, the samples were concentrated using standard sedimentation technique described by Hansen and Perry [23]. Briefly, about 3 g of faeces were taken in to centrifuge tube and 40 ml of water was added and then mixed thoroughly. The suspension was filtered through a tea strainer into other centrifuge tube and was left for 15 min. Thereafter, the supernatant was decanted and the sediment was re-suspended. This step was repeated 3 times until the supernatant was cleared. Finally, the sediment was transferred with a pipette to a clean slide and observed under low power (10x) microscope. The slides were judged positive when oval to spindle shaped with centrally bulged and terminal spine on one side of egg was identified.

### Data analysis

Data was entered into Microsoft Excel spread sheets and analysed using SPSS statistical software version 16. Descriptive statistics were computed to determine frequency and percentage. Chi-square test was used to analyse associations between independent variables (such as cattle origin, breed, sex, age group and body condition) and BS infection. Variables that independently showed significant association were entered in to a logistic regression model. The magnitude of association was measured using 95% confidence interval Odds Ratio (B Exp). Logistic regression model assumptions were tested by changing significance of the Change in − 2 Log Likelihood at *p*-value of < 0.05.

The study was reviewed and approved by College of Veterinary Medicine - Jigjiga University. Verbal informed consent was obtained from cattle owners to involve animals in the study. Thereafter, stool sample were collected with no harm caused on the cattle as per the research ethics of the college.

## Results

Out of the total 360 faecal samples examined, 80 (22.2%) were found to be positive for *S.bovis* eggs. Higher BS prevalence was recorded in Ahurie kebele followed by Afrefida kebele which constituted 40 and 26.7% respectively. The disease showed higher infection rate in local breed (25.9%) than in cross breed (13.3%). High infection rates was observed in females and in the age group 2–3 years with the prevalence of 25.6 and 26.4% respectively. The prevalence of BS was high (36.1%) in cattle with poor condition (Table 1).

**Table 1:** The prevalence of Bovine Schistosomiasis based on cattle origin, breed, sex, age and body condition in the study area

Variable	Catagory	Total examined	No. of positive (%)	χ^2^	*P*-value
Origin	Ahurie	90	36(40.0)	32.4	0.000
	Afrefida	90	24(26.7)		
	Kar	90	14(15.6)		
	Gedema	90	6(6.7)		
Breed	Local	255	66 (25.9)	6.78	0.009
	Cross	105	14(13.3)		
Sex	Male	122	19(15.6)	4.72	0.030
	Female	238	61(25.6)		
Age	< 2	98	18(18.4)	2.22	0.329
	2–5	129	34(26.4)		
	> 5	133	28(21.1)		
Body condition	Poor	133	48(36.1)	26.4	0.000
	Medium	111	21(18.9)		
	Good	116	11(9.5)		

As depicted in Table 2, cattle origin followed by body condition were identified as major factors associated with prevalence of BS (*P* = 0.000). These were followed by the breed and sex of cattle (*P* < 0.05). Cattle in Ahurie kebele were 3.162 times more likely to be infected by BS than cattle in Gedema kebele whereas cattle in Kar kebele had 0.18 times less chance of acquiring BS infection. Cattle with poor body condition were 7.045 times more likely to harbour BS than cattle with good body condition. Female cattle were 1.923 times at high risk of BS infection than male cattle whereas local breed cattle were 2.558 times more likely to be infected by BS than cross breed cattle.

**Table 2:** Binary logistic regression model output for factors associated with infection rate of Bovine Schistosomiasis in the study area

Variable	Catagory	Positive (%)	B	*Exp (B)*	95% CI	*P*-value	Change in −2 Log Likelihood	*P*-value
Origin	Ahurie	36(40.0)	1.151	3.162	1.497–6.680	0.003	46.128	0.000
	Afrefida	24(26.7)	−0.365	0.694	0.308–1.566	0.379		
	Kar	14(15.6)	−1.675	0.187	0.069–0.507	0.001		
	Gedema	6(6.7)						
Breed	Local	66(25.9)	0.939	2.558	1.282–5.102	0.008	7.904	0.005
	Cross	14(13.3)						
Sex	Male	19(15.6)					4.281	0.039
	Female	61(25.6)	0.654	1.923	1.018–3.634	0.044		
Body condition	Poor	48(36.1)	1.952	7.045	3.171–15.652	0.000	30.718	0.000
	Medium	21(18.9)	0.767	2.154	0.938–4.948	0.070		
	Good	11(9.5)						

## Discussion

Epidemiological studies on animal schistosomiasis that focus on identifying target population and epidemiological mechanisms including factors determining transmission and spread are key for designing appropriate disease control strategies in endemic areas. The prevalence of BS in the current study was lower than previous reports from the same district [15]. This may be attributed to the fact that only random lottery method selected local breed cattle were investigated in the latter study. Meanwhile, comparable [11, 13, 16, 24, 25], lower [8, 10, 24, 26] and higher [21, 27] BS prevalence rates were previously reported from different regions of the country. Corresponding trends may be attributed to study design, agro-ecological and husbandry related differences.

Infection rate of BS was significantly varied across different localities of the study district. This might be related to differences in topography and agro-ecological factors, such as temperature, moisture, humidity, availability of large permanent water bodies, and irrigation practices crucial to sustain schistosome life cycle. Proximity of cattle residence and grazing area to cercaria infested water bodies can be an important factor underlying observed prevalence variations. Hansen and Perry [23] reported that key determinant in the epidemiology of bovine schistosomiasis is the relative abundance of the intermediate hosts and their ability to develop and survive in the environment which is associated with large permanent water bodies. Marshy areas and stagnant water bodies like small streams, ponds, and swamps around rivers, lakes and irrigation sites represent major sources of infection for schistosomiasis [5].

Currently, BS affected local breed cattle more than cross-breeds. This trend was consistent with other studies [13, 28] but in conflict of other findings [14, 24, 25, 27]. Variations observed between Fogeral and Fogera - Holstein Frisian crossbred cattle is most probably attributed to difference of husbandry conditions under which corresponding are maintained. Cross-bred dairy cattle are commonly kept under semi/ intensive management system which reduce risk of exposure to infected reservoir hosts. However, investigations are required to rule out potential genetic basis of predisposition to BS.

In agreement with current findings, some researchers had reported a higher prevalence of BS in female cattle compare to in male cattle [13, 24, 28] whereas others noted a reverse trend [10, 29, 30]. In the present study area, female cattle commonly grazed in *Schistosoma* contaminated pastures and water points whereas male cattle spent most of their time on farm land ploughing activities which could be reducing risk of exposure to cercaria infested habitats. However, the role of sex hormone related immunological influences on differential disease susceptibility trends also demands further study. On the other hand, a higher prevalence of BS was noted in cattle exhibiting poor nutritional status. This is in agreement with findings reported by Merawe et al. [30] and Belayneh and Tadesse [11]. Urqhart et al. [5] and Hansen and Perry [23] had reported that bovine schistosomes utilized the hosts’ nutrition and compromise its immune competence, both of which lead to pathologies, including anorexia, emaciation and disease predisposition. In this study, a relatively (*p* > 0.05) higher prevalence of BS was noted in adult cattle. A similar trend was also reported by Melkamu [31] in Bahir Dar and the surrounding areas. Lower risk of BS in younger animals could be attributed to husbandry differences which reduce risk of environmental exposures. On the other hand, old cattle may have developed stronger acquired immunity which suppresses worm fecundity and the release of parasitic eggs in faeces [32].

## Conclusion

BS is a common disease problem of cattle in South Achefer district. Prevalence of BS showed significant variations across study kebele’s, cattle breeds and sexes as well as a negative association with nutritional performance. Such variations emphasize critical relevance of considering agro-ecological conditions and husbandry practice related risk factors in disease prevention and control inventions. Potential genetic and physiological (sex hormone related) influences need to be ruled out through deeper investigation.

## Abbreviations

BS: Bovine schistosomiasis
CI: Confidence interval
OR: Odds ratio
